# Prognostic value of preoperative hematological markers in patients with glioblastoma multiforme and construction of random survival forest model

**DOI:** 10.1186/s12885-023-10889-0

**Published:** 2023-05-12

**Authors:** Xiaozong Duan, Bo Yang, Chengbin Zhao, Boran Tie, Lei Cao, Yuyuan Gao

**Affiliations:** The First Affiliated Hospital of Zhengzhou University, Zhengzhou, China

**Keywords:** Glioblastoma multiforme, Prognostic analysis, Preoperative hematological markers, Inflammatory immune index, Random survival forest model

## Abstract

**Objective:**

In recent years, an increasing number of studies have revealed that patients’ preoperative inflammatory response, coagulation function, and nutritional status are all linked to the occurrence, development, angiogenesis, and metastasis of various malignant tumors. The goal of this study is to determine the relationship between preoperative peripheral blood neutrophil to lymphocyte ratio (NLR), monocyte to lymphocyte ratio (MLR), systemic immune-inflammatory index (SII), platelet to lymphocyte ratio (PLR), and platelet to fibrinogen ratio (FPR). Prognostic nutritional index (PNI) and the prognosis of glioblastoma multiforme (GBM) patients, as well as establish a forest prediction model that includes preoperative hematological markers to predict the individual GBM patient’s 3-year survival status after treatment.

**Methods:**

The clinical and hematological data of 281 GBM patients were analyzed retrospectively; overall survival (OS) was the primary endpoint. X-Tile software was used to determine the best cut-off values for NLR, SII, and PLR, and the survival analysis was carried out by the Kaplan–Meier method as well as univariate and multivariate COX regression. Afterward, we created a random forest model that predicts the individual GBM patient’s 3-year survival status after treatment, and the area under the curve (AUC) is used to validate the model’s effectiveness.

**Results:**

The best cut-off values for NLR, SII, and PLR in GBM patients’ preoperative peripheral blood were 2.12, 537.50, and 93.5 respectively. The Kaplan–Meier method revealed that preoperative GBM patients with high SII, high NLR, and high PLR had shorter overall survival, and the difference was statistically significant. In addition to clinical and pathological factors. Univariate Cox showed NLR (HR = 1.456, 95% CI: 1.286 ~ 1.649, *P* < 0.001) MLR (HR = 1.272, 95% CI: 1.120 ~ 1.649, *P* < 0.001), FPR (HR = 1.183,95% CI: 1.049 ~ 1.333,* P* < 0.001), SII (HR = 0.218,95% CI: 1.645 ~ 2.127, *P* < 0.001) is related to the prognosis and overall survival of GBM. Multivariate Cox proportional hazard regression showed that SII (HR = 1.641, 95% CI: 1.430 ~ 1.884, *P* < 0.001) is also related to the overall survival of patients with GBM. In the random forest prognostic model with preoperative hematologic markers, the AUC in the test set and the validation set was 0.907 and 0.900, respectively.

**Conclusion:**

High levels of NLR, MLR, PLR, FPR, and SII before surgery are prognostic risk factors for GBM patients. A high preoperative SII level is an independent risk factor for GBM prognosis. The random forest model that includes preoperative hematological markers has the potential to predict the individual GBM patient’s 3-year survival status after treatment,and assist the clinicians for making a good clinical decision.

## Introduction

Patients with GBM have a poor overall prognosis, with an overall survival time of only 12–15 months after surgery plus STUPP [1]. As a result, accurately predicting the prognosis of GBM patients is critical. According to research, the prognosis of GBM patients is affected by their age, tumor characteristics, treatment plan, and other factors [2]. However, the accuracy of predicting the prognosis of GBM is still limited, and more prognostic factors are needed to evaluate the prognosis of GBM patients. Preoperative systemic inflammatory response, coagulation function, and nutritional status of patients have all been shown in studies to influence the anti-tumor effect [3–6]. Some preoperative hematological markers, such as NLR, MLR, PLR, FPR, SII (SII = NLR* platelet count), and PNI (PNI = albumin + 5* lymphocytes), have been linked to the prognosis of certain malignant tumors, including gastric cancer, esophageal cancer, colorectal cancer, and breast cancer [7–12]. However, there is no agreement on the role of preoperative hematological markers in GBM, and more research is required.

The overall survival time of GBM patients can be improved after surgery plus the STUPP protocol, but the difference in prognosis and survival time between patients remains significant [13]. It is critical to screen out these patients with poor prognoses and accurately predict patient survival time after treatment.

In this study, the prognostic factors of 281 GBM patients treated at the first affiliated hospital of Zhengzhou University were systematically examined. Our goals are as follows: 1. to investigate the predictive value of preoperative peripheral blood inflammatory response, coagulation function, and nutritional status in patients with GBM. 2. Develop a GBM random forest prognosis model that incorporates clinical fundamentals, molecular pathology, imaging features, and preoperative peripheral blood markers. At the same time, we collected data on 115 GBM patients treated at the People’s Hospital of Henan Province to further validate the forecast model.

## Materials and methods

### Research data

Collect data on GBM patients who were admitted to the Department of Neurosurgery at Zhengzhou University’s First Affiliated Hospital between 2015 and 2018, and received surgical treatment as well as regular postoperative radiotherapy and chemotherapy. 281 patients were included based on the inclusion and exclusion criteria. (1) Patients who underwent surgery for the first time in our hospital’s neurosurgery department were diagnosed with GBM by pathology after surgery and completed the “STUPP” radiotherapy and chemotherapy regimen; (2) age was 18 years old; (3) hematological examination within 1 week before the operation; (4) head magnetic resonance spectroscopy imaging one week before the operation. (5) and patients with complete follow-up data. Exclusion criteria include (1) preoperative puncture biopsy, radiotherapy, and chemotherapy; (2) infectious diseases; (3) severe heart, lung, liver, and kidney disease; (4) auto-immune disease; (5) poor magnetic resonance imaging quality; and (6) severe intra-tumor hemorrhage.

Using the same criteria and methods, data of 115 patients treated for GBM in Henan Provincial People’s Hospital were collected for validation of the prediction model. This investigation followed the Helsinki Declaration and was approved by the Ethics Committee of the First Hospital of Zhengzhou University.

### Basic clinical data

Patients who meet the enrollment criteria should have their clinical basic data recorded and collected in detail, including their age, gender, intracranial hypertension, epilepsy, preoperative KPS score, and tumor resection degree. Criteria for Tumor resection degree: All patients were reexamined with skull-enhanced MRI 48–72 h after surgery, and the calculation formula for tumor resection degree was (preoperative tumor volume–postoperative residual tumor volume)/preoperative tumor volume. Resection greater than or equal to 95% was defined as total resection, Less than 95% resection was defined as incomplete resection.

### Hematological data

Fasting hematology indices, including neutrophil count, monocyte count, lymphocyte count, platelet count, serum albumin concentration, plasma fibrinogen, and other indices, were collected within one week before surgery for all patients who met the enrollment criteria. Determine the NLR, MLR, PLR, FPR, PNI, and SII values.

### Imaging data

All patients in our hospital underwent an examination by MRS within a week of the operation. MRS examination results from eligible patients were collected, which included tumor location, maximum diameter of the tumor, single or sporadic tumor, N-acetylaspartic acid (NAA) value, choline (Cho) value, and creatine (Cr) value in the tumor area, and NAA/Cr, Cho/Cr, and Cho/NAA ratios.

### Molecular and immunohistochemistry results

Patients’ postoperative pathological data were reviewed, and the expression levels of IDH mutant or wild type, p53, and Ki67 proteins were meticulously recorded. The IDH classification is based on the 2016 edition of the World Health Organization Classification of Central Nervous System Tumors. Ki67 was expressed using a percentile system, with 30% indicating low expression and 30% indicating high expression. The p53 protein was expressed in “-to +  +  +  + ”, “- ~  + ” was low expression; “ +  + ” and above was high expression.

### Follow-up method

Outpatient reexamination, phone inquiries, and medical records were used to obtain follow-up data once every three months in the first year after surgery and once every six months beginning in the second year after surgery, with death as the end point of follow-up. The total survival time (TST) is defined as the patient’s survival time from the date of operation to death or the last follow-up. The follow-up time of this group ranged from 3.5 to 63 months, with a median follow-up time of 19 months. In total, 213 (75.8%) patients reached the endpoint. The deadline to follow up is August 31, 2021.

### Statistical method

IBM SPSS26.0 software was used for statistical analysis. The mean standard deviation (x s) is used between the two groups, the t-test is used between the two groups, one-way ANOVA is used for comparison among multiple groups, and the rest are expressed by median (interquartile spacing), and a nonparametric test is used for comparison. 0.05 is the test level. The best cutoff values for NLR, MLR, PLR, FPR, PNI, SII, NAA/Cr, Cho/Cr, and Cho/NAA were determined using the ROC curve. The Kaplan–Meier method and the Log Rank test were used to assess patients’ postoperative survival. The univariate and multivariate Cox proportional hazard regression models were used to calculate the risk ratio (HR) and 95% confidence interval (CI), and the influence was determined. To create a multivariate Cox regression forest map, we used the R package forest plot.

### Construction of random forest prognostic model

Patients’ data are grouped and labeled (training set = 0, test set = 1), and the selected variables are organized into a data matrix, with each row representing a patient and each column representing a variable. The prognosis prediction model in this study is built using the R language R package random Forest. The required R language function package is first loaded, followed by the sorted data matrix being read into the R language program and divided into a training set and a testing set based on the data grouping labels (training set = 0, testing set = 1). The random survival forests prediction model will then be trained in the training set. The receiver’s working curve (ROC) can be used to assess the predictive model’s ability. The area under the curve (AUC) ranges from 0 to 1. The larger the AUC, the better the model’s predictive ability. Finally, we further verified the predictive ability of the model on the verification set.

## Results

### Data characteristics of patients

Patients were divided into two groups based on whether they died at the end of the follow-up period (214 cases) or survived (67 cases). At the end of follow-up, the age (*P* < 0.001), KPS score (*P* = 0.002), preoperative NLR (*P* < 0.001), MLR(*P* = 0.001), PLR(*P* = 0.001) and SII (*P* < 0.001) of GBM patients between the two groups. Other data differences were not statistically significant (*P* > 0.05) (See Table 1).

**Table 1:** Data of two groups of patients with different outcomes

Variable	Death group (*n* = 214)	Survival group (*n* = 67)	χ^2^/t/Z	*P* value
Age > 65 years old [example (%)]	56(26.2%)	2(3%)	-	< 0.001
Male [example (%)]	119(55.6%)	36(53.7%)	0.073	0.788
Intracranial hypertension [cases (%)]	136(63.6%)	40(59.7%)	0.323	0.570
Epilepsy [cases (%)]	64(29.9%)	20(29.8%)	0.001	0.993
Kps < 70 [example (%)]	143(66.8%)	58(86.5%)	9.768	0.002
Total tumor resection [cases (%)]	162(75.7%)	54(80.6%)	0.688	0.407
NLR	2.60(2.01,3.40)	1.88(1.41,2.31)	5.853	< 0.001
MLR	0.28(0.21,0.36)	0.21(0.19,0.30)	3.178	0.001
PLR	138(108,175)	116(88.5,160)	3.415	0.001
FPR	76(62,110)	75(59,89.5)	1.737	0.082
PNI	51.0(48.1,55.0)	51.4(49.0,52.7)	0.401	0.689
SII	652.5(474,802)	406(278,511)	7.723	< 0.001
Frontotemporal lobe [example (%)]	60(28.8%)	45(67.2%)	33.377	< 0.001
Maximum diameter ≥ 5 cm[ example (%)]	73(34.1%)	27(40.3%)	0.852	0.356
Single tumor [cases (%)]	106(49.5%)	34(50.7%)	0.030	0.862
NAA/Cr	0.67(0.30,1.00)	0.73(0.44,1.02)	1.538	0.124
Cho/Cr	2.15(1.71,3.41)	1.96(1.72,3.20)	0.855	0.392
Cho/NAA	4.08(1.77,10.00)	3.48(1.77,5.26)	1.697	0.090
IDH mutant (example,%)	23(10.7%)	36(53.7%)	92.364	< 0.001
Low expression of Ki67 (e.g.,%)	151(70.5%)	44(67.2%)	-	1.000
Low expression of p53 (e.g.,%)	184(28.8%)	49(28.8%)	5.946	0.015

### Univariate Cox survival analysis

Initially, we systematically evaluated the prognostic value of each factor in patients with GBM. Univariate Cox regression analysis showed that age (*P* < 0.001, HR = 3.43), tumor location (*P* < 0.001, HR = 0.421), KPS (*P* < 0.001, HR = 0.975), NLR (*P* < 0.001, HR = 1.456), FPR(*P* = 0.006, HR = 1.183), SII (*P* < 0.001, HR = 1.871) and IDH (*P* < 0.001, HR = 0.218) were significantly correlated with the overall survival of GBM (See Table 2).

**Table 2:** Cox regression analysis of prognostic factors of glioblastoma multiforme by single factor forward introduction method

Variable	B	SE	Wald	df	Sig	HR	95.0% CI for HR
Gender	-0.064	0.138	0.213	1.000	0.644	0.938	0.716 ~ 1.230
Age	1.113	0.162	47.273	1.000	0.000	3.043	2.216 ~ 4.179
Intracranial hypertension	-0.003	0.143	0.000	1.000	0.985	0.997	0.751 ~ 1.319
Preoperative epilepsy	0.183	0.152	1.459	1.000	0.227	1.201	0.751 ~ 1.319
KPS score	-0.025	0.006	17.167	1.000	0.000	0.975	0.963 ~ 0.987
Tumor resection degree	0.078	0.161	0.238	1.000	0.626	1.082	0.0789 ~ 1.482
NLR	0.376	0.063	35.011	1.000	0.000	1.456	1.286 ~ 1.649
MLR	0.241	0.065	13.615	1.000	0.000	1.272	1.120 ~ 1.649
PLR	0.265	0.064	17.125	1.000	0.000	1.303	1.150 ~ 1.477
PNI	-0.102	0.064	2.547	1.000	0.111	0.903	0.797 ~ 1.024
FPR	0.168	0.061	7.529	1.000	0.006	1.183	1.049 ~ 1.333
SII	0.626	0.066	91.004	1.000	0.000	1.871	1.645 ~ 2.127
Tumor site	-0.864	0.154	31.636	1.000	0.000	0.421	0.312 ~ 0.569
Maximum diameter of tumor	0.014	0.060	0.057	1.000	0.812	1.014	0.902 ~ 1.140
Solitary	-0.024	0.137	0.032	1.000	0.859	0.976	0.746 ~ 1.276
NAA/Cr	-0.050	0.059	0.720	1.000	0.396	0.951	0.846 ~ 1.068
Cho/Cr	-0.001	0.060	0.000	1.000	0.987	0.999	0.888 ~ 1.123
Cho/NA	0.113	0.061	3.441	1.000	0.064	1.119	0.994 ~ 1.261
IDH	-1.525	0.222	47.084	1.000	0.000	0.218	0.141 ~ 0.336
Ki67	0.147	0.096	2.345	1.000	0.126	1.158	0.960 ~ 1.397
p53	0.076	0.066	1.316	1.000	0.251	1.079	0.948 ~ 1.228

### Kplan-Meier survival curve

According to age 65, the KPS score was 70, and the IDH status was classified as wild type or mutant type. Using the ROC curve, the optimal NLR, PLR, and SII cutoff values were determined to be 2.12, 93.5, and 537.5, respectively. The Log-Rank test revealed the statistical significance of the difference between survival curves.

**Fig. 1:**
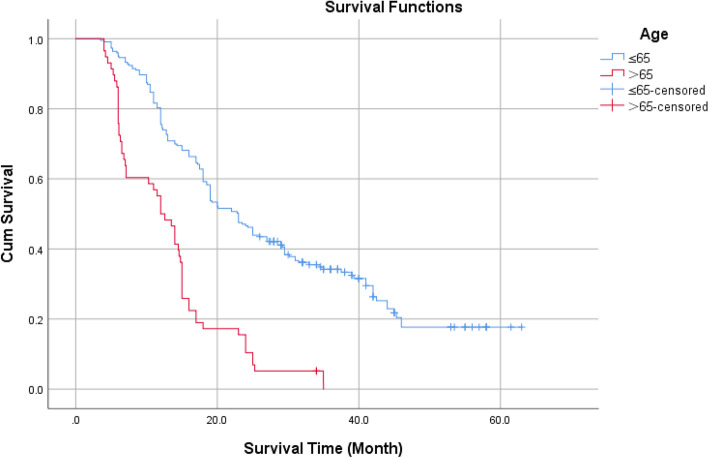
Cumulative survival rate of different age groups without endpoint events

Kaplan–Meier survival curve analysis showed that the cumulative survival rate of patients aged ≤ 65 years was higher than that of Patients aged > 65 years old (Fig. 1), The log-rank test showed that the difference is statistically significant with *P* < 0.05.

**Fig. 2:**
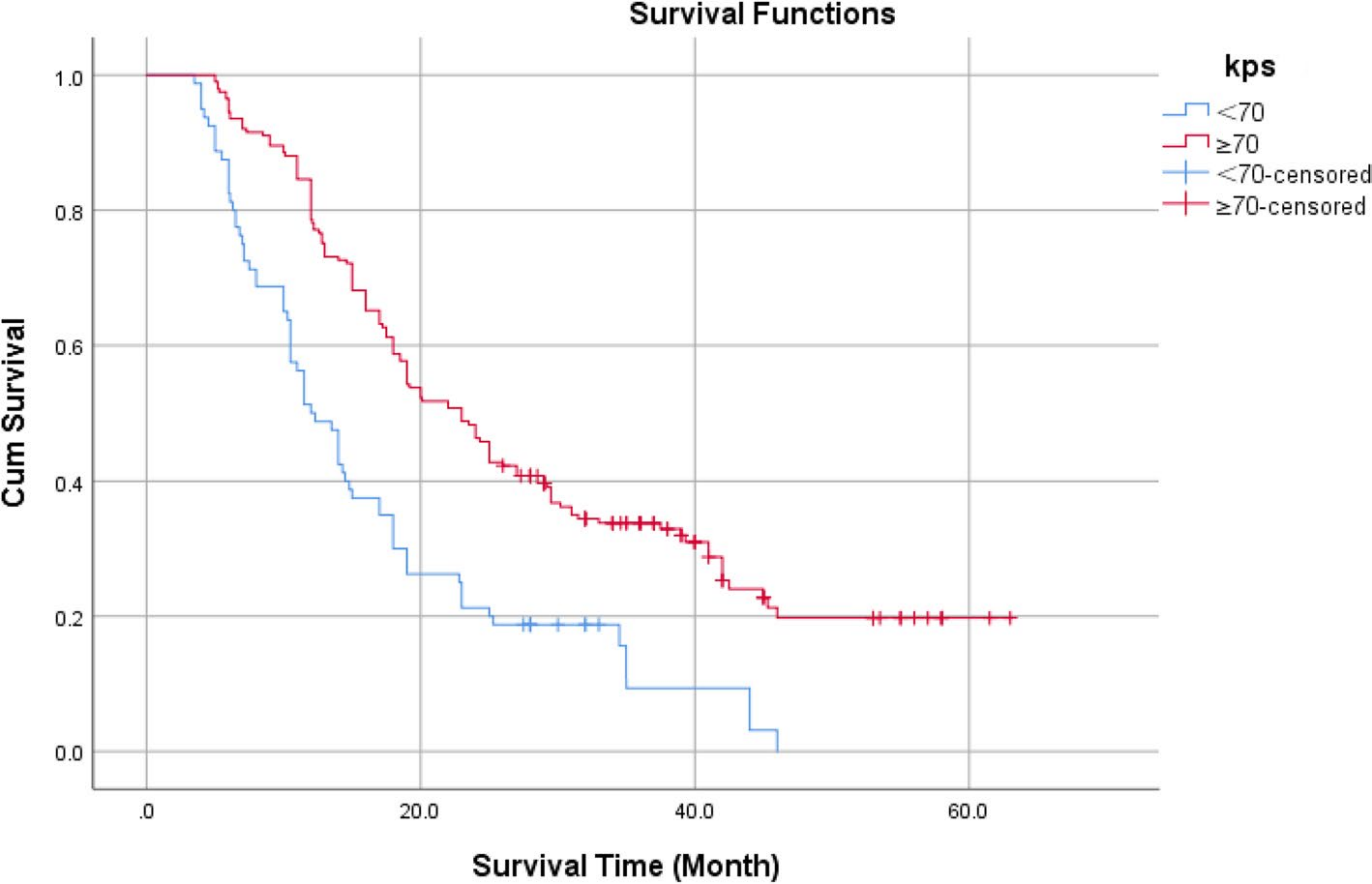
Cumulative survival rate of different KPS levels without end-point events

Kaplan–Meier survival curve analysis showed that the cumulative survival rate of patients with KPS score ≥ 70 was higher than that of patients with KPS score < 70 (Fig. 2), the log-rank test showed that the difference is statistically significant with *P* < 0.05.

**Fig. 3:**
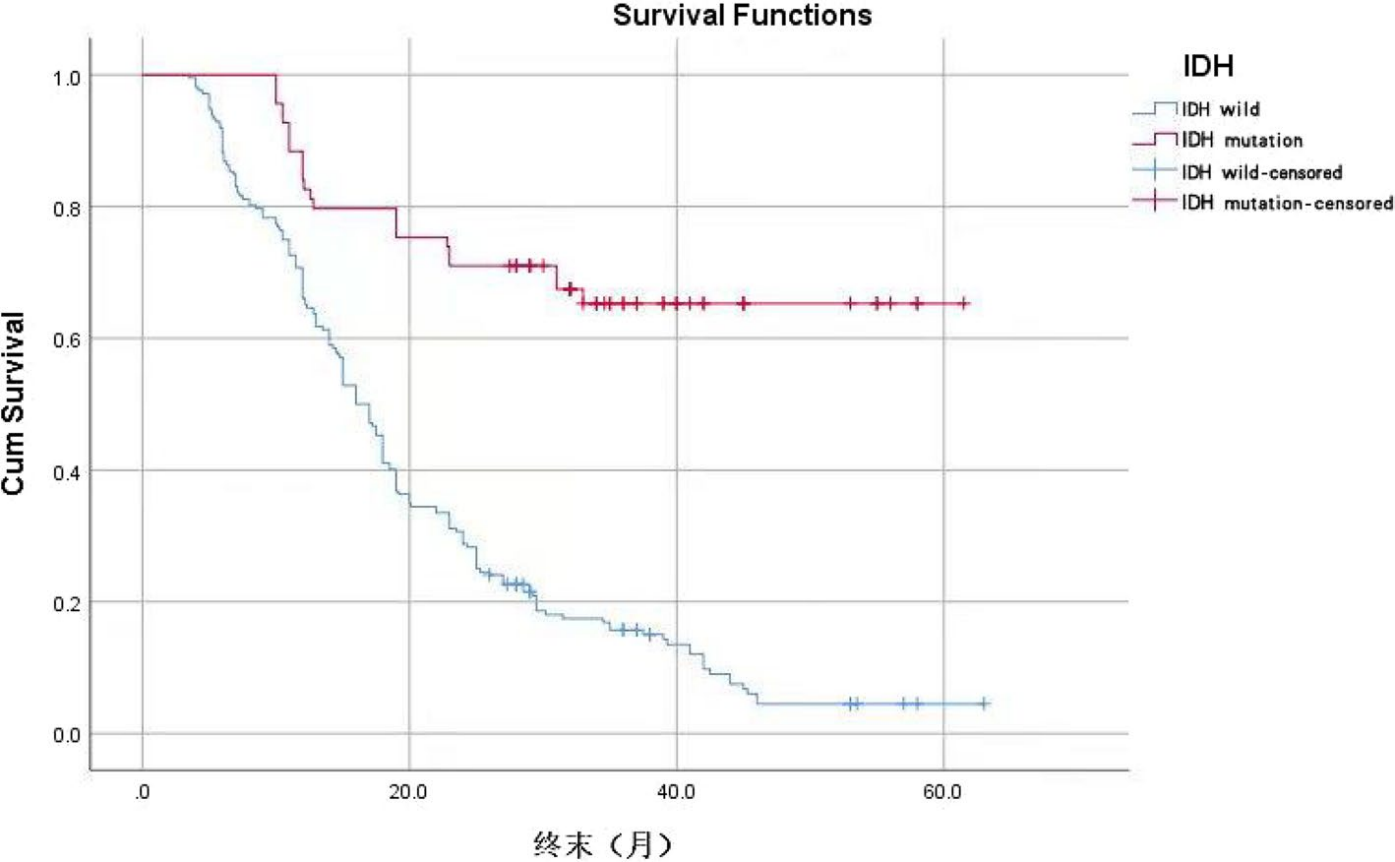
Cumulative survival rate of different IDH states without endpoint events

Kaplan–Meier analysis of survival curves revealed that the cumulative survival rate of patients with IDH mutation was greater than that of patients with IDH wild (Fig. 3), the log-rank test showed that the difference is statistically significant with *P* < 0.05.

**Fig. 4:**
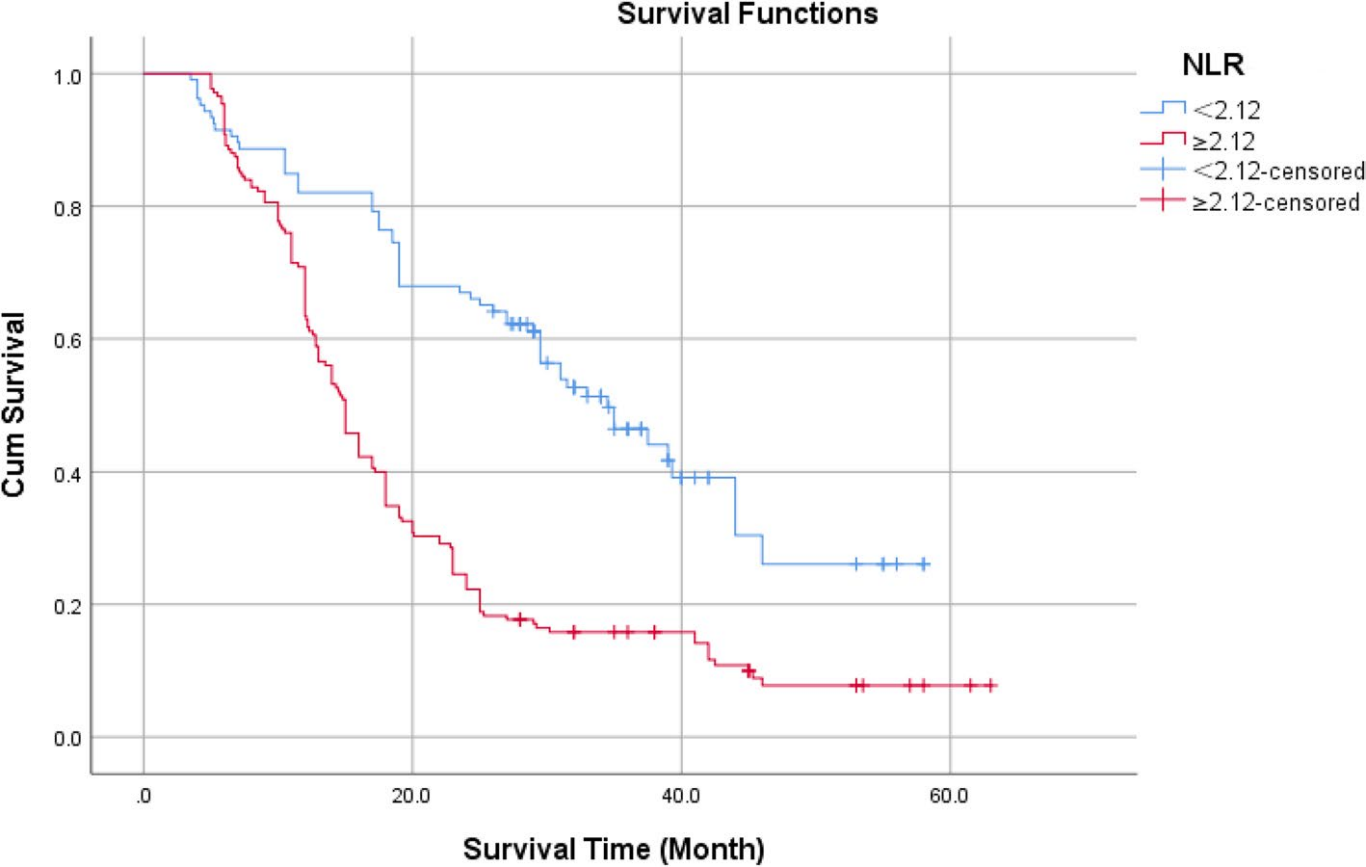
Cumulative survival rate of different levels of NLR without endpoint events

Kaplan–Meier survival curve analysis showed that the cumulative survival rate of patients with NLR < 2.12 was higher than that of Patients with NLR ≥ 2.12 (Fig. 4), the log-rank test showed that the difference is statistically significant with *P* < 0.05.

**Fig. 5:**
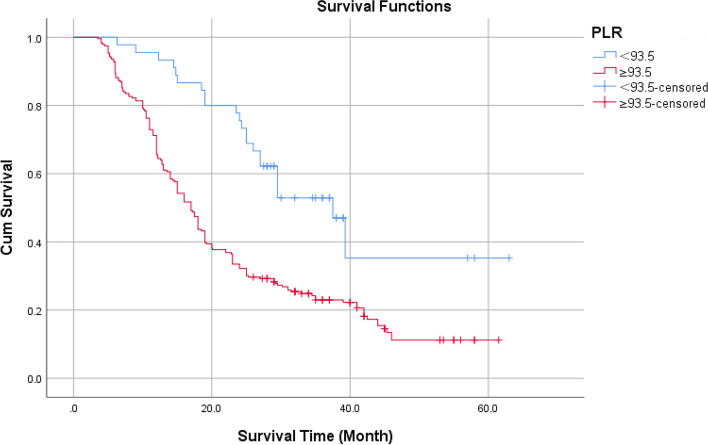
Cumulative survival rate of different levels of PLR without endpoint events

Kaplan–Meier survival curve analysis showed that the cumulative survival rate of patients with PLR < 93.5 was higher than that of Patients with PLR ≥ 93.5 (Fig. 5), the log-rank test showed that the difference is statistically significant with *P* < 0.05.

**Fig. 6:**
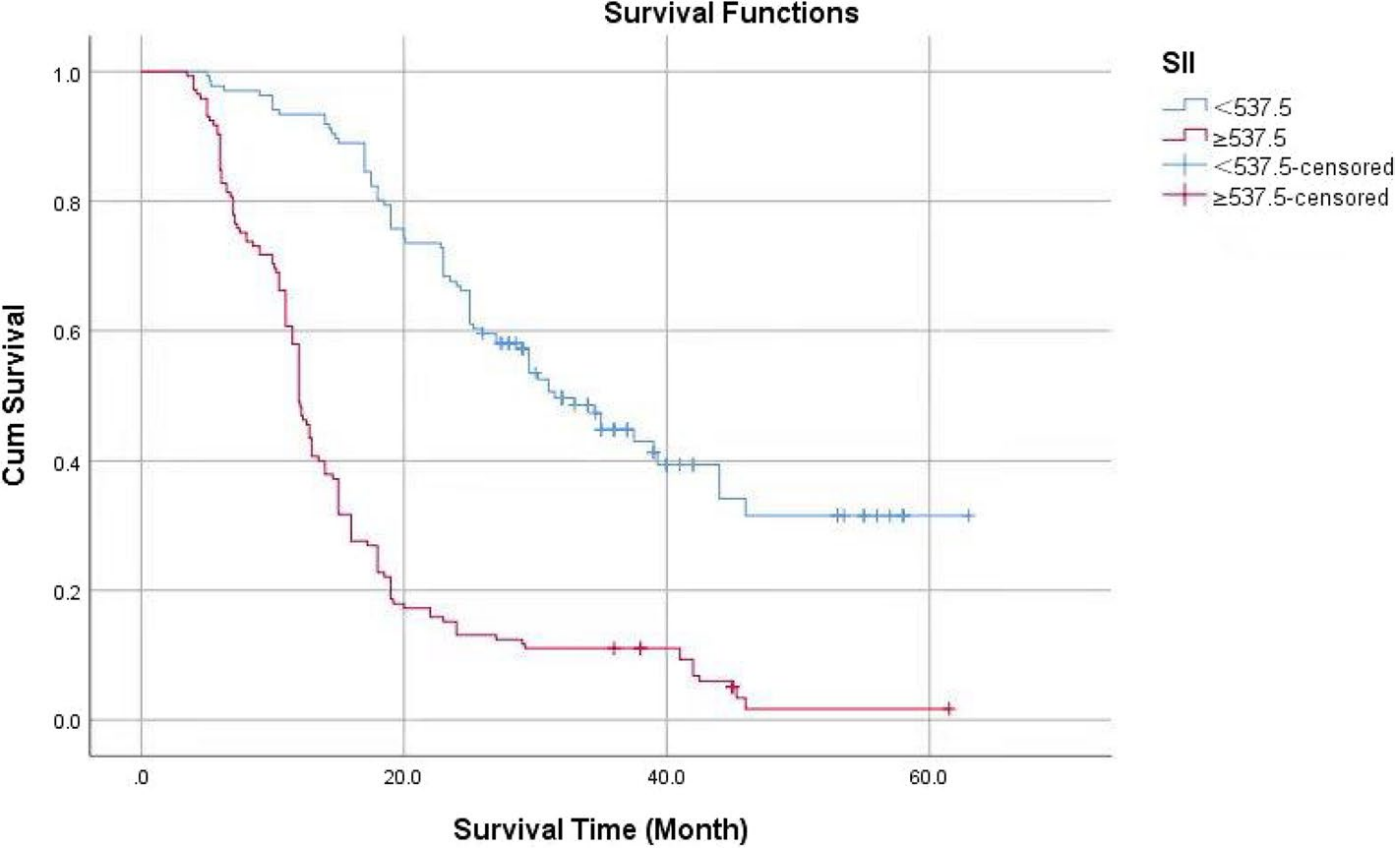
Cumulative survival rate of different levels of SII without endpoint events

Kaplan–Meier survival curve analysis showed that the cumulative survival rate of patients with SII < 537.5 was higher than that of Patients with SII ≥ 537.5 (Fig. 6), The log-rank test showed that the difference is statistically significant with *P* < 0.05.

### Multivariate Cox survival analysis

**Fig. 7:**
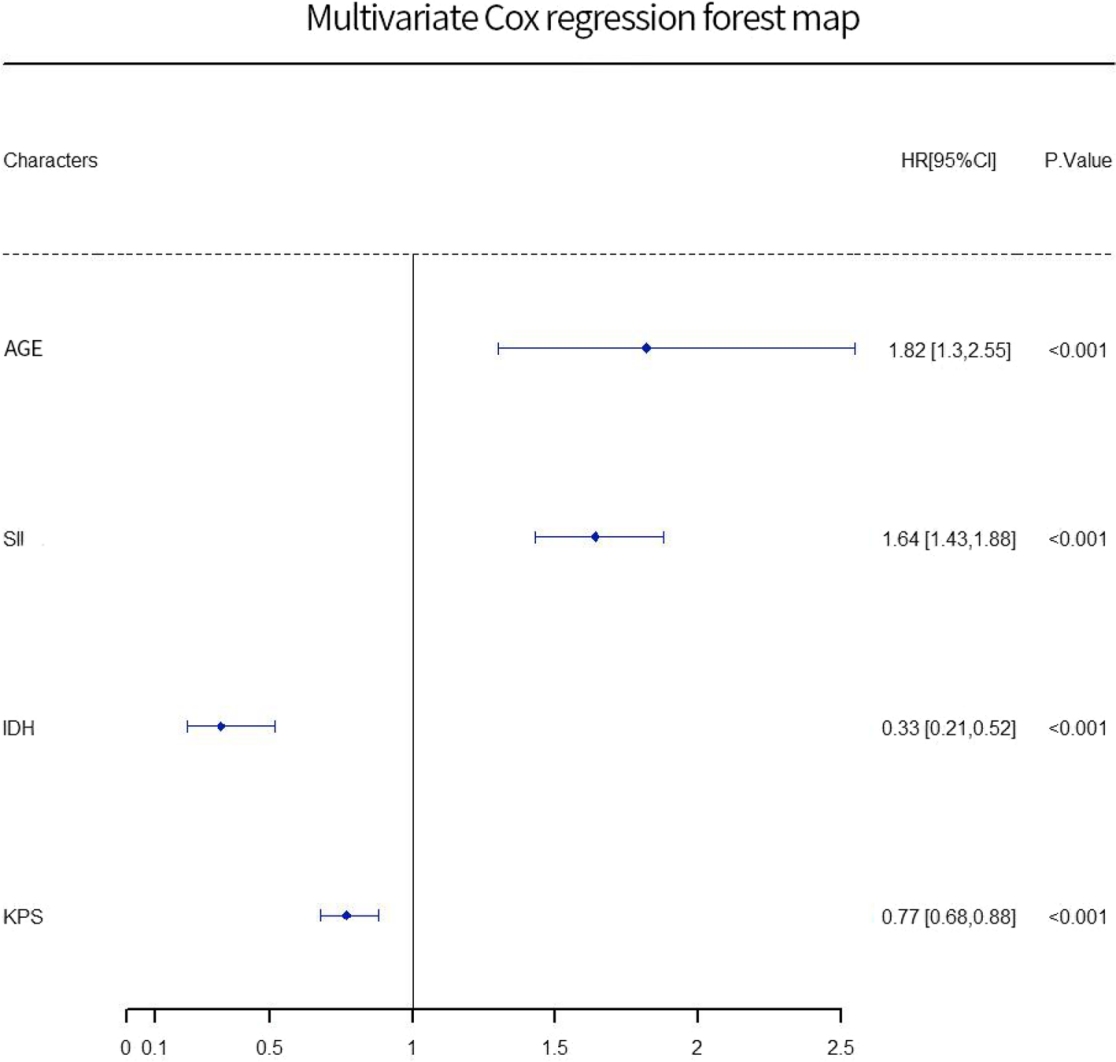
Cox regression forest diagram of prognosis of glioblastoma multiforme

Further multivariate Cox regression analysis and the establishment of multivariate Cox regression forest diagram showed that age > 65 years old (*P* < 0.001, HR = 1.823) and SII ≥ 537.5 (*P* < 0.001, HR = 1.641) were risk factors related to the survival of GBM patients (Table 3, Fig. 7). GBM patients with IDH mutation (*P* < 0.001, HR = 0.327) and *KPS* score ≥ 70 (*P* < 0.001, HR = 0.775) tend to have a better survival prognosis.

**Table 3:** Cox regression analysis of prognostic factors of glioblastoma multiforme by multi-factor forward introduction method

Vriable	B	SE	Wald	df	Sig	HR	95.0% CI for HR
Age	0.600	0.171	12.349	1.000	0.000	1.823	1.304 ~ 2.547
SII	0.495	0.070	49.573	1.000	0.000	1.641	1.430 ~ 1.884
IDH	-0.118	0.232	23.199	1.000	0.000	0.327	0.207 ~ 0.515
KPS	-0.255	0.065	15.474	1.000	0.000	0.775	0.682 ~ 0.880

### Construction and evaluation of random forest prediction model

Two hundred and eighty-one GBM patients were randomly divided into a training set (*n* = 168) and a testing set (*n* = 113), with the training set being used to train the random forest model and the test set being used to evaluate the prediction system’s accuracy. The trained random forest model used in binary tree has three variables (mtry), and it contains five hundred decision trees (ntree). The model’s accuracy is 100% for in the training set and 92.92% in the testing set. The prediction model was further validated using the 115 patients treated for GBM at Henan People’s Hospital as the validation set, with an accuracy rate of 93.91% (Table 4).

**Table 4:** Confusion matrix of random forest model

Training set		Predicted value	
		die	survive
True value	die	124	0
	survive	0	44
Test set		Predicted value	
		die	survive
True value	die	85	5
	survive	3	20
Validation set		Predicted value	
		die	survive
True value	die	88	3
	survive	4	20

**Fig. 8:**
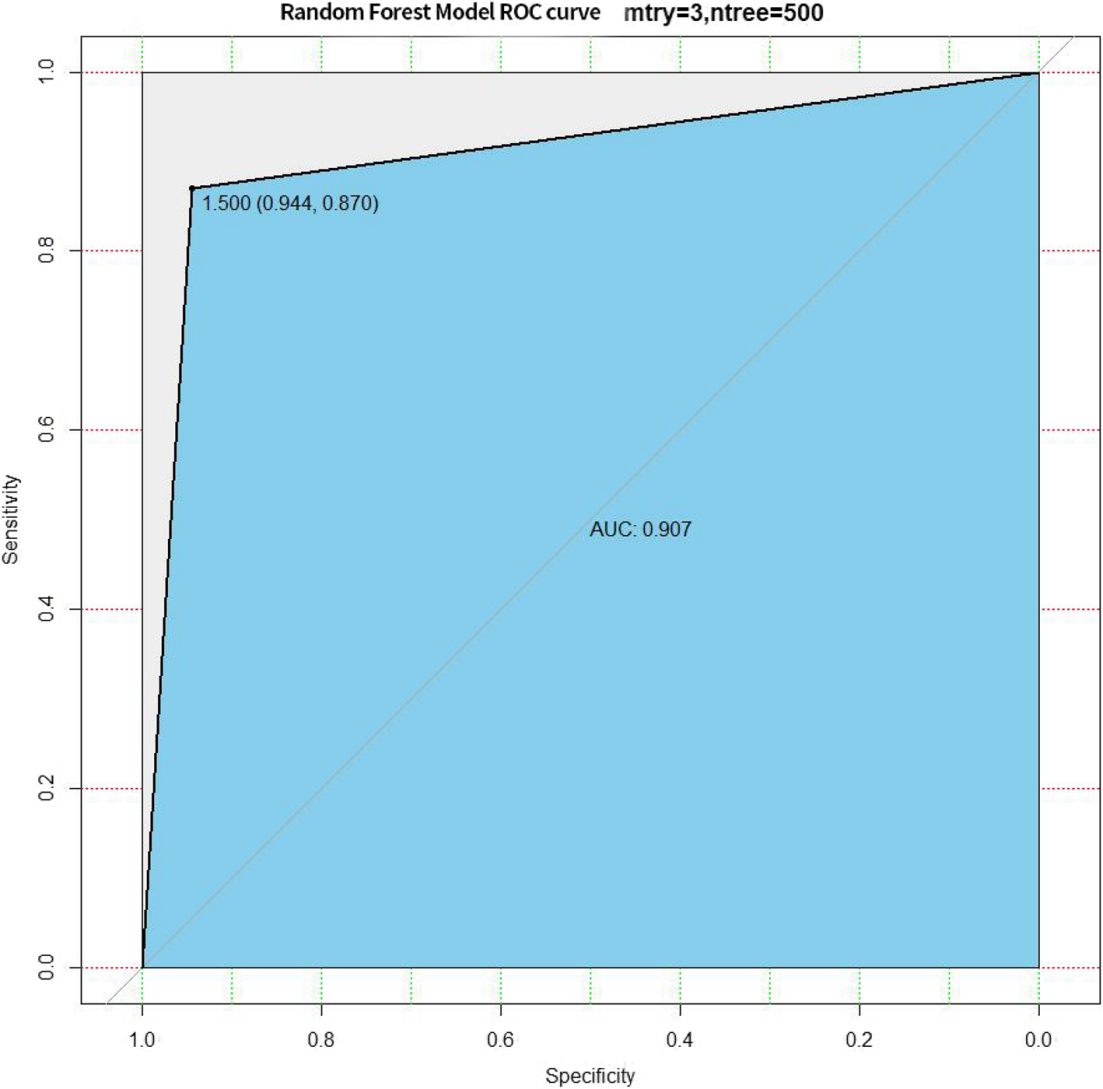
The receiver operating characteristic curve of the random forest model using the testing set

**Fig. 9:**
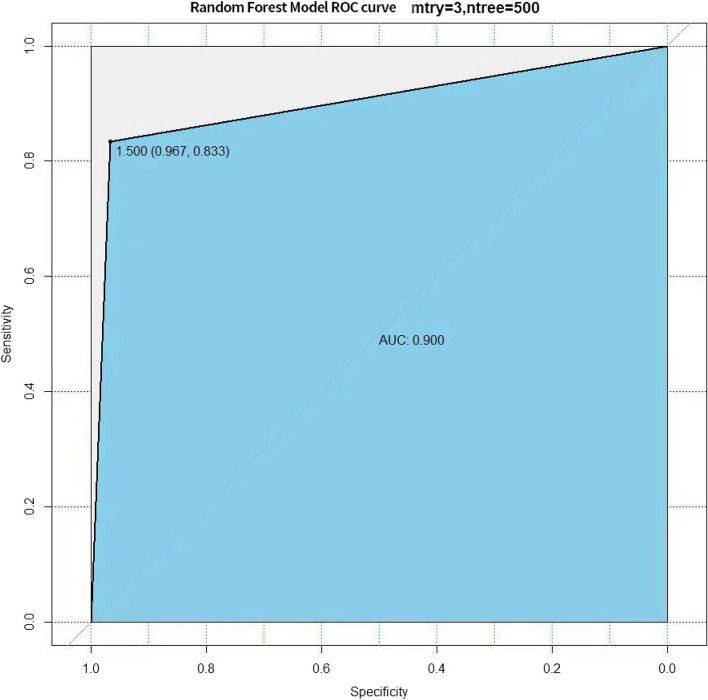
The receiver operating characteristic curve of the random forest model using the validation set

The random forest model’s receiver operating characteristic curve was plotted using the testing set prediction results, and the model had a sensitivity of 0.870 and a specificity of 0.944, with an AUC of 0.907 (Fig. 8). While using the validation set prediction results, it had a sensitivity of 0.833 and a specificity of 0.967, with an AUC of 0.900 (Fig. 9).

### Variable weight of the random forest model

**Fig. 10:**
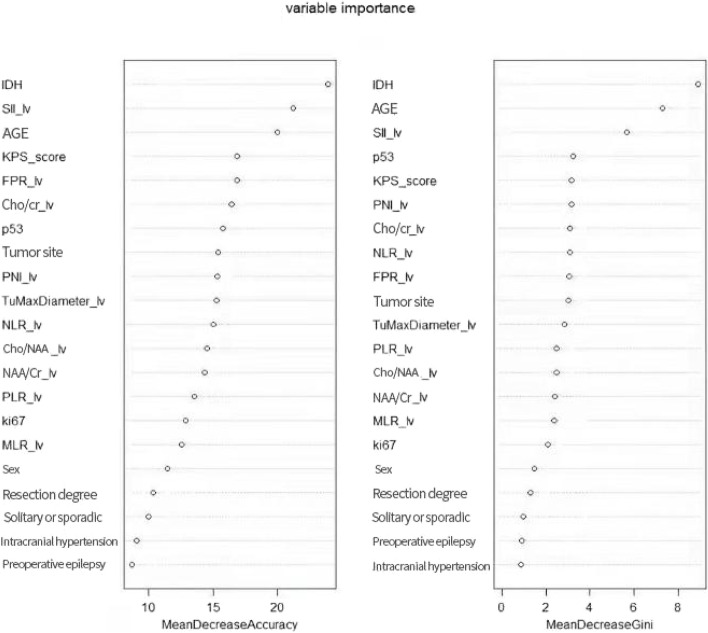
Variable weight ranking of random forest model

The random Forest R package is used to examine the random forest model’s variable weights. Mean Decrease Accuracy represents the percentage of random disruption of a feature. The higher the accuracy of the model, the more important the feature is. This is used to calculate the relative importance of variables. The top five variables influencing the importance of prognosis in GBM patients are IDH, SII, age, KPS, and FPR. Likewise, Mean Decrease Gini employs the Gini index to calculate the relative importance of features. IDH, age, SII, p53, and KPS are the top five variables that influence the importance of prognosis in GBM patients (See Fig. 10).

## Discussion

An accurate prognosis evaluation is critical for GBM treatment design and clinical management. However, current prognosis prediction models are unable to meet the medical needs of GBM patients. This study systematically examined the clinical features, molecular pathological features, magnetic resonance spectroscopy imaging features, and prognostic value of the preoperative peripheral blood inflammation index, coagulation index, and nutritional status index of 281 patients with GBM. A random forest prognosis model with peripheral blood markers was developed. This model accurately predicts GBM patients’ 3-year survival after surgical resection and the + STUPP regimen.

Cancer-related inflammation has emerged as a new cancer indicator in recent decades. Nutritional status, inflammatory status, and immune function are frequently thought to be related to the prognosis in patients with malignant tumors [14, 15]. Inflammation, as part of the tumor microenvironment, can promote tumor occurrence, development, angiogenesis, and metastasis, as well as affect tumor patients’ clinical outcomes. As a result, inflammatory cells in peripheral blood can interact with tumor cells directly or indirectly, promoting tumors’ malignant biological behavior [16]. Inflammatory conditions, such as pro-inflammatory cytokines, growth factors, and chemokines, directly contribute to cancer progression [17]. The tumor microenvironment also generates inflammatory mediators that contribute to cell apoptosis and angiogenesis [6, 18]. We discovered that preoperative NLR, MLR, PLR, FPR, and SII were associated with the prognosis of GBM patients who received surgical treatment plus the STUPP protocol. As a result, a high level of SII was an independent prognostic factor of GBM. Reasons for analysis: Neutrophils have the potential to alter the tumor microenvironment by secreting angiogenic growth factors (such as vascular endothelial growth factors and matrix metalloproteinases) and inhibiting the cytotoxic activity of other immune cells (such as activated T cells, natural killer cells, and so on) [19]. Lymphocytes, on the other hand, inhibit tumor progression and eliminate new tumor cells as part of anti-cancer immunity [20]. Monocytes and their highly specialized macrophages inhibit tumor cell migration, invasion, metastasis, tumor-related angiogenesis, and the anti-tumor immune response [21]. Monocytes will be recruited into the brain parenchyma as a source of tumor-associated macrophages under any pathological condition, particularly glioma [22]. Platelet-tumor aggregates are responsible for the recruitment of neutrophils and the release of factors related to tumor growth, metastasis, and angiogenesis during the first few hours of cancer cell colonization. Some studies, on the other hand, have found that platelet-transmitted factors can have cytotoxic effects on proliferating tumor cells and even enhance apoptosis [23]. As a result, inflammatory cells in the peripheral blood can interact with tumor cells directly or indirectly, promoting tumors’ malignant biological behavior. The SII is a comprehensive index that combines neutrophil, lymphocyte, and platelet counts and can indicate the level of inflammation in cancer patients [24]. Thrombocytopenia, neutropenia, and/or lymphopenia are frequently present in glioma patients, along with an increase in SII in peripheral blood, all of which can promote tumor cell differentiation, proliferation, and metastasis [25, 26].

A prognostic model is a model that predicts the outcome of diseases. It is commonly used in the clinic to predict disease progression, patient survival time, and the likelihood of developing a specific disease stage. It employs a variety of predictive factors and methods. Many researchers have attempted similar studies on the prognosis evaluation model for glioma patients. Rathore et al. [27] determined the likelihood of predicting the early recurrence of GBM after surgery using the radiochemical characteristics of edema around the tumor. This model can help guide super-total resection and/or postoperative intensive radiotherapy. Wang et al. [28] performed univariate and multivariate Cox regression analysis on GBM and human autophagy-related genes in the American Cancer Gene Atlas Database (TCGA). The genes neuroregulatory protein 1, integrin subunit 3, and microtubule-binding protein 1 light chain 3 were chosen to create a prognostic risk scoring model, and a prognostic nomogram was created that included autophagy characteristics, age, drug therapy, radiotherapy, and the IDH mutation. This model was proven useful. Gorlia et al. [29] developed a prognosis model of GBM patients treated with temozolomide based on age, surgical scope, the mini-mental state evaluation score, and MGMT methylation status, which was used to assess the factors influencing GBM patient survival. Some researchers developed a glioma prognosis model based on non-coding RNA and divided patients into high-risk and low-risk groups [30]. Peng et al. [31] conducted a thorough examination of PDI family members and discovered several new potential signal pathways involved in the progression of glioma. A glioma prognosis nomogram was developed based on the survival risk score of PDIs and other clinical factors. These studies have fully validated the prognostic evaluation model’s positive role in predicting patient prognosis. In recent years, an increasing number of molecular markers have been used to predict the prognosis of gliomas, but the research and development cycle for these markers is lengthy and expensive, preventing widespread application.

Based on the foregoing, this study gathered easily accessible and low-cost clinical data, hematological data, magnetic resonance imaging data, pathological and immunohistochemical data, and built a random forest prognosis model of GBM after surgical resection and the STUPP regimen. In the testing set and validation set, our prediction model’s sensitivity (0.870, 0.833), specificity (0.944, 0.967), and AUC (0.907, 0.900) respectively. Because of the small sample size, there is some over-fitting in the training set, resulting in a 100% accuracy rate. The reason for selecting this model is that it has the following advantages over other machine learning methods and traditional mathematical models: 1. it can be used for a wide range of data types. 2. It can handle numerous input variables. 3. It can assess the importance of variables. 4. It can maintain accuracy even when data is lost, and 5. It is faster to learn.

Without a doubt, this study has some limitations: 1. this is a retrospective study, the number of patients included is limited, and the follow-up time is inadequate. 2. Patients may receive different treatments after surgery, such as bevacizumab, and the duration of treatment may vary. This may cause research findings to deviate. More prospective clinical trials are needed to assess the prognostic value of hematological indicators in GBM patients. 3. The GBM random forest prognosis model developed in this study requires additional external validation.

## Conclusion

High levels of NLR, MLR, PLR, FPR, and SII before surgery are prognostic risk factors for GBM patients. A high preoperative SII level is an independent risk factor for GBM prognosis. The random forest model that includes preoperative hematological markers has the potential to predict the individual GBM patient’s 3-year survival status after treatment, and assist the clinicians for making a good clinical decision.

## Data Availability

The datasets used and/or analyzed during the current study are available from the corresponding author upon reasonable request.
